# Pericoronary Adipose Tissue as Storage and Supply Site for Oxidized Low-Density Lipoprotein in Human Coronary Plaques

**DOI:** 10.1371/journal.pone.0150862

**Published:** 2016-03-24

**Authors:** Yasumi Uchida, Yasuto Uchida, Ei Shimoyama, Nobuyuki Hiruta, Toshihiko Kishimoto, Soichiro Watanabe

**Affiliations:** 1 Japanese Foundation for Cardiovascular Research, Funabashi, Japan; 2 Department of Cardiology, Tokyo Jikei University School of Medicine, Tokyo, Japan; 3 Department of Cardiology, Tsukuba Memorial Hospital, Tsukuba, Japan; 4 Department of Pathology, Funabashi-Futawa Hospital, Funabashi, Japan; 5 Department of Pathology, Toho University Sakura Medical Center, Sakura, Japan; 6 Department of Biomolecular Science, Faculty of Science, Toho University, Okubo, Funabashi, Japan; 7 Department of Biomolecular Science, Faculty of Science, Toho University, Okubo, Funabashi, Japan; Nagoya University, JAPAN

## Abstract

**Objectives:**

It is generally believed that low-density lipoprotein enters the vascular wall from its lumen and oxidized (oxLDL), after which it plays an important role in atherosclerosis. Because voluminous epicardial adipose tissue is a risk factor for coronary events, there is a possibility that the pericoronary adipose tissue (PCAT), which is a part of epicardial adipose tissue, acts as a risk factor by supplying oxLDL to the coronary arterial wall. The present study was performed whether PCAT stores and supplies oxLDL to the coronary wall.

**Methods:**

Localization of oxLDL in PCAT and its relation to plaque morphology were examined by immunohistochemical techniques in 27 epicardial coronary arteries excised from 9 human autopsy cases.

**Results:**

OxLDL deposited in all PCAT of the studied cases. The percent (%) incidence of oxLDL in the intima of 25 normal segment, 19 white plaques, 15 yellow plaques without necrotic core (NC) and 10 yellow plaques with NC, was 32, 84, 93 (p<0.05 vs normal segments and yellow plaques with NC), and 30, respectively. OxLDL deposited either in dotted or diffuse pattern. Double immunohistochemical staining revealed that the dotted oxLDL was that contained in CD68(+)-macrophages. The oxLDL-containing macrophages were observed in the interstitial space but not inside of the vasa vasorum, and they traversed PCAT, adventitia, external and internal elastic laminae, suggesting their migration towards the intima. Diffuse oxLDL deposits were observed in 17 preparations, the majority of which were co-localized with the vasa vasorum in outer or in both inner and outer halves of intima, and rarely in the inner half alone.

**Conclusions:**

The results suggested that PCAT is a supply source of oxLDL to coronary intima and acts as a risk factor for coronary events, that oxLDL increasingly deposits in the intima with plaque growth and decreases after plaque maturation, and therefore molecular therapies targeting the PCAT before plaque growth could be effective in preventing human coronary atherosclerosis.

## Introduction

It is generally believed that low-density lipoprotein (LDL) enters the vascular wall from the lumen and is oxidized (oxLDL), after which it plays a key role in the initiation, progression and destabilization of atherosclerotic plaques [1–3], and monocytes migrate into the vascular wall from the lumen, and become macrophages [4, 5], that in turn accumulate oxLDL and become foam cells, while producing collagen-degrading enzymes such as metalloproteases and collagenases which destroy collagen fibers, and also hypochlorous acid (OHCl) which damages endothelial cells, resulting in vulnerable plaques [6–10].

Till date, it is the sole known process of oxLDL generation in vascular wall and it is not known whether there are other mechanisms for oxLDL deposition in the vacular wall in man.

Because voluminous epicardial adipose tissue is a risk factor for coronary events [11], there is a possibility that the pericoronary adipose tissue (PCAT), which is a part of epicardial adipose tissue adjacent to the coronary artery, acts as a risk factor by supplying oxLDL to the adjacent coronary arterial wall. The PCAT releases a number of cytokines which influence coronary function [12] but it is not known whether or not it stores and releases oxLDL to the adjacent coronary arterial intima which is the site of atherosclerosis.

Using immunohistochemical techniques, the present study was performed to examine whether and how human PCAT stores and supplies oxLDL to the coronary artery.

## Methods

### 1. Angioscopic and Immunohistochemical Studies of Excised Human Pericoronary Adipose Tissue (PCAT) and Its Adjacent Coronary Artery

#### (1). Ethical statement

This ex vivo study was carried out after approval of the Ethical Committees of the Japan Foundation for Cardiovascular Research, Funabashi-Futawa Hospital and Toho University, and after obtaining written informed consent from the families concerned on the use of excised coronary artery and its surrounding adipose tissue for angioscopic and histological studies to clarify the mechanisms of atherosclerosis.

#### (2). Subjects

The 27 proximal to middle segments of coronary arteries (9 left anterior descending arteries, 9 left circumflex arteries, 9 right coronary arteries) and the surrounding adipose tissue were carefully excised from 9 successive cases of patients who had died and underwent autopsy from April 1, 2010 to June 30, 2015 at Funabashi-Futawa Hospital or Toho University Medical Center Sakura Hospital [61 ± 3.6 years; 3 females, 6 males; acute myocardial infarction (3), diabetic nephropathy (3), cerebral infarction (1), subarachnoid hemorrhage (1) and sudden death (1)] (Table 1).

**Table 1:** Backgrounds of Autopsy Cases.

Patients	Age/sex	Disease	Cause of death	Medication
TT	46/F	Subarachnoid	Do	Emergency
		hemorrhage		Treatment
AR	48/F	Sudden death	Do	Emergency
				Treatment
YM	58/M	Acute myocardial	CHF	PCI, nitrates, diuretics,
		infarction		clopidogrel, etc
UI	60/M	Acute myocardial	Pneumonia	CABG
		infarction		
MM	60/M	Diabetic nephropathy	Pneumonia	Hemodyalysis
ST	63/M	Diabetic nephropathy	Sudden death	Insulin, hypertensives
JS	72/F	Acute myocardial	Bleeding from	PCI, nicorandil,
		infarction	gastric cancer	clopidogrel, etc
KB	73/M	Cerebral infaction	Do	Emergency
				Treatment
NS	78/M	Diabetic nephropathy	Colon cancer	Insulin, pitavastatin,
				Etc

CHF: congestive heart failure. PCI: percutaneous coronary intervention. CABG: coronary artery bypass grafting.

#### (3). Classification of coronary plaques and normal segments by conventional angioscopy

In the present study, a conventional coronary angioscopy system (details described elsewhere [13]) was used to classify coronary plaques and normal segments.

#### (4). Plaques and normal segments

By conventional coronary angioscopy, plaque was defined as a nonmobile, protruding or lining mass clearly demarcated from the adjacent normal wall and with a shape, location and color that did not alter after saline flushing. A normal segment was defined as a milky-white and smooth-surfaced portion without any protrusions [14]. Classification of non-disrupted plaques and normal segments was performed independently using images recorded on DVD disks by two observers who did not participate in conventional angioscopy. Disrupted plaques were excluded because thrombi and plaque debri might disturb plaque color assessment and immunohistochemical staining.

#### (5). Plaque color assessment

Because color definition differs observer to observer, influenced by their experience and visual sense, we developed a more objective method for color definition. Plaque images obtained by conventional angioscopy were classified as white or yellow by an AquaCosmos image analyzer (C7746, Hamamatsu Photonics, Hamamatsu, Japan). Based on the relationship between color spectrum generated through a prism by a Xenon lamp and its light wavelength, the light wavelength of “yellow” was defined as between 575 and 595 nm. The color band of the area being imaged through an angioscope was separated by the image analyzer into three primary colors, namely red, green and blue. The intensity ratio of red: green: blue of the “yellow” color was 1.0: 1.38–1.46: 0.47–0.60. Similarly, white light generated by the Xenon lamp was separated into three primary colors with an intensity ratio of 1.0: 0.9–1.1: 0.9–1.1, which defined “white” plaque[15].

### 2. Observation of Excised Coronary Arteries by Conventional Angioscopy

A Y-connector was introduced into the proximal portion of the respective coronary artery for perfusion with saline solution at a rate of 20 mL/min and then the angioscope was introduced through the connector into the artery to evaluate it for plaques. A total of 19 white plaques, 25 yellow plaques and 25 normal segments, which matched aforementioned criteria were confirmed by conventional angioscopy in 27 coronary arteries. Externally, the location of each observed plaque or normal segment could be identified because the light irradiated by the angioscope tip was visible through the arterial wall.

#### (1). Selection of plaques and normal segments

The 4–5-mm long section of artery in which the observed plaque was located and its surrounding PCAT were isolated by transecting its proximal and distal ends at the shorter axes. Normal segments were similarly isolated. Plaques and normal segments which that had not been damaged by the manipulation were selected for immunohistochemical analysis. In total, 19 white plaques (1 each from 19 arteries), 25 yellow plaques 1 each from 25 arteries), and 25 normal segments (1 each from 25 arteries), surrounded by PCAT were selected, and embedded in O.C.T. Compound (Sakura Fintek USA Inc., Torrance, CA) before being stored at -20°C.

### 3. Immunohistochemistry of OxLDL, CD68(+)-macrophages and CD31 (Endothelial Cells of Vasa Vasorum)

#### (1). Definition of PCAT

Epicardial adipose tissue which located within 3 mm (nearly the same as the diameter of the proximal to middle segments of a coronary artery) of the external elastic lamina of an epicardial coronary artery was arbitrarily defined as PCAT because it may more likely to directly influence the coronary artery than epicardial adipose tissue located remotely, based on the observation of microvessels penetrating from the PCAT to the epicardial coronary arterial wall was observed within a few mm from coronary arterial wall [16].

#### (2). Immunohistochemical staining

All plaques and normal segments with their surrounding EPCAT, which had been stored at -20°C, were cut into successive 20 μm sections on a cryostat (Tissue Tec 3D, SakuraFinetec Japan, Tokyo). Such relatively thick and frozen sections were used to prevent any substances leaking from the PCAT. Next, the sections were fixed with 4% paraformaldehyde solution for 7 min at 4°C, incubated with a mixture of 1% hydrogen peroxide in methanol for 30min. The successive sections were stained immunohistochemically in the order of oxLDL, CD68 for macrophages and CD31 for endothelial cells of vasa vasorum (arising from the mother vessel and/or penetrating microvessels arising from PCAT [17]. Briefly, a section was reacted with anti-oxLDL antibody (Anti-oxLDL-antibody orb 10973; rabbit polyclonal, which reacts with human oxLDL; Biorbyt Ltd, Cambridge, UK) diluted to 100-fold (μg/mL) for 60 min, the peroxidase reaction was developed by 3,3’-diaminobenzidine tetrahydrochloride using Envision kit (Code No K4061,DAKO Co, Grostrup, Denmark) for 30 min, and finally the cell nuclei were stained with hematoxylin. The next section was pretreated similarly, reacted with anti-CD68 antibody mouse monoclonal NCL-CD 68-KP1; antigen lysosomal granules from human lung macrophages; Leica Biosystems Newcastle Ltd, Newcastle, UK) diluted to 200-fold, and was treated similarly to stain macrophages [18, 19]. The third section was reacted with anti-CD31-antibody (rabbit polyclonal; immunogen: synthetic peptide corresponding to C terminals of mouse CD31; reacts with mouse and human CD31; Abcam Co, Tokyo, Japan) diluted to 50-fold, reacted with biotinylated anti-goat IgG diluted to 100-fold, and treated similar to oxLDL to stain CD31 in the endothelial cells of vasa vasorum [20]. Using these immunohistochemical techniques, all relevant molecules were stained brown.

#### (3). Double immunohistochemical staining

Double staining of oxLDL and CD68 was performed to confirm that oxLDL-containing mononuclear cells were macrophages. Briefly, after washing with phosphate-buffered saline, a section was reacted with anti-CD68 antibody as described before for 60 min, and with anti-mouse Alexa555 (Alexa Fluoro555 goat anti-mouse IgG, Code A21422, Molecular Probe Ltd, CA, USA) for 30 min to elicit the red fluorescence of CD68. The same section was the reacted with anti-oxLDL antibody for 60 min, and with anti-rabbit FITC (FITC conjugated AffeiniPureoat Anti-Rabbit IgG, Code 111-095-003, Vector Laboratories Inc, Burlingame, CA, USA) for 30 min to elicit the green fluorescence of oxLDL. Finally, the section was reacted with DAPI (4’,6-diamidino-2-phenylindole; Life Technologies Carlsbad, Carlsbad, CA, USA) to elicit blue fluorescence of cell nuclei [21]. Also, M2-macrophages were stained with Alexa-labelled anti-CD206 antibody (Human MMR/CD206 antibody, Catalog no. AF2534, source: polyclonal goat IgG, immunogen: mouse myeloma cell line NSO-derived recombinant human MMR/CD206, reacts with human, R&D Systems Ltd, Minneapolis, MN, USA) [22, 23] and double stained with oxLDL. OxLDL, macrophages and their nuclei thus stained were photographed separately or merged with one over another through a confocal laser scanning microscope (FLUOVIEW FV 1700; Olympus Co, Tokyo, Japan) using a 460 nm band-pass filter (BPF) and a 510 nm band absorption filter (BAF) to obtain the green fluorescence of oxLDL, a 555 nm BPF and a 575 nm BAF to obtain the red fluorescence of macrophages, and a 345 nm BPF and 420 nm BAF to obtain the blue fluorescence of cell nuclei.

### 4. Microscopic Observation of PCAT and Coronary Artery after Immunohistochemical Staining

#### (1). Definition of the deposition pattern of oxLDL

Histological observation of the deposition of oxLDL was performed using a microscope (IX 70, Olympus Co., Tokyo, Japan). It was classified as dotted (i.e. pencil-tip-like small points with diameter ≤30 μm) or diffuse (diameter ≥100 μm). We considered the dotted deposition was significant if the dot density was ≥5 /200 × 200 μm^2^, because a small number of dots were disseminated not only in the intima but also in the PCAT in the majority of sections stained for oxLDL.

#### (2). Relationship between plaque morphology and oxLDL deposition

Percentage (%) deposition of oxLDL (both dotted and diffuse patterns) in PCAT, adventitia, and intima was compared among normal coronary segments, white plaques and yellow plaques with and without necrotic core (NC). Deposition in the intima (including plaque) was further classified as inner layer (luminal side) deposition or outer layer (medial side).

#### (3). Relationship between oxLDL-containing macrophages in the intima and PCAT

Using double-immunohistochemically stained preparations, the relationship between the density of oxLDL-containing macrophages in the intima and PCAT was examined to understand the origin of oxLDL-containing macrophages in the intima.

#### (4). Relationship between intimal vasa vasorum and oxLDL deposition

By staining with CD31, a marker of vascular endothelial cells, we examined whether the deposition of oxLDL in the intima is dependent on the formation of intimal vasa vasorum, which penetrates the adventitia (penetrating microvessels) into the intima.

### 5. Statistical Analysis

The data obtained were tested by Fisher’s exact test. A value of p< 0.05 was considered to be statistically significant.

## Results

### 1). Storage of OxLDL in PCAT

**Fig 1:**
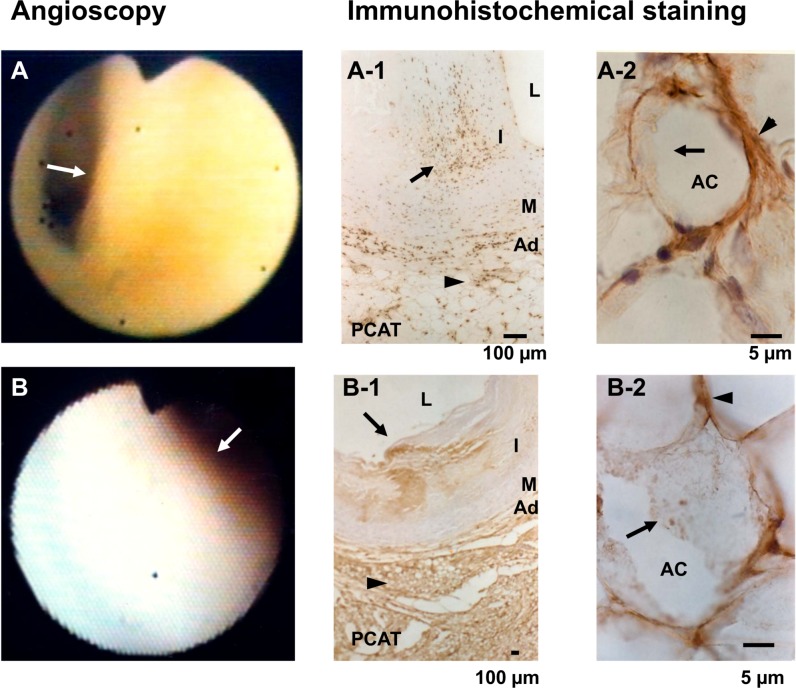
Localization and deposition patterns of oxidized low-density lipoprotein (oxLDL) in human pericoronary adipose tissues (PCAT) and coronary arterial wall. (A). Yellow plaque by angioscopy. By immunohistochemical staining, the plaque (arrow in A) contained dotted oxLDL deposits (arrow in A-1) and diffuse or dotted oxLDL deposits in PCAT (arrowhead in A-1). By magnifying the PCAT, it became clear that oxLDL deposited not only in the cytoplasm (arrow in A-2) but also in plasma membrane (arrowhead in A-2). (B). White plaque by angioscopy. By immunohistochemical staining, diffuse deposition of oxLDL was observed in the intima (arrow in B-1) and PCAT (arrowhead in B-1). On magnification, oxLDL deposited in cytoplasm (arrow) and plasma membrane (arrowhead in B-2). AC: adipocyte, L: lumen, I: intima, M: media, and Ad: adventitia, in this and following figures. Scale bars in A-1, B-1 = 100μm. Scale bars in A-2, B-2 = 5μm.

**Fig 2:**
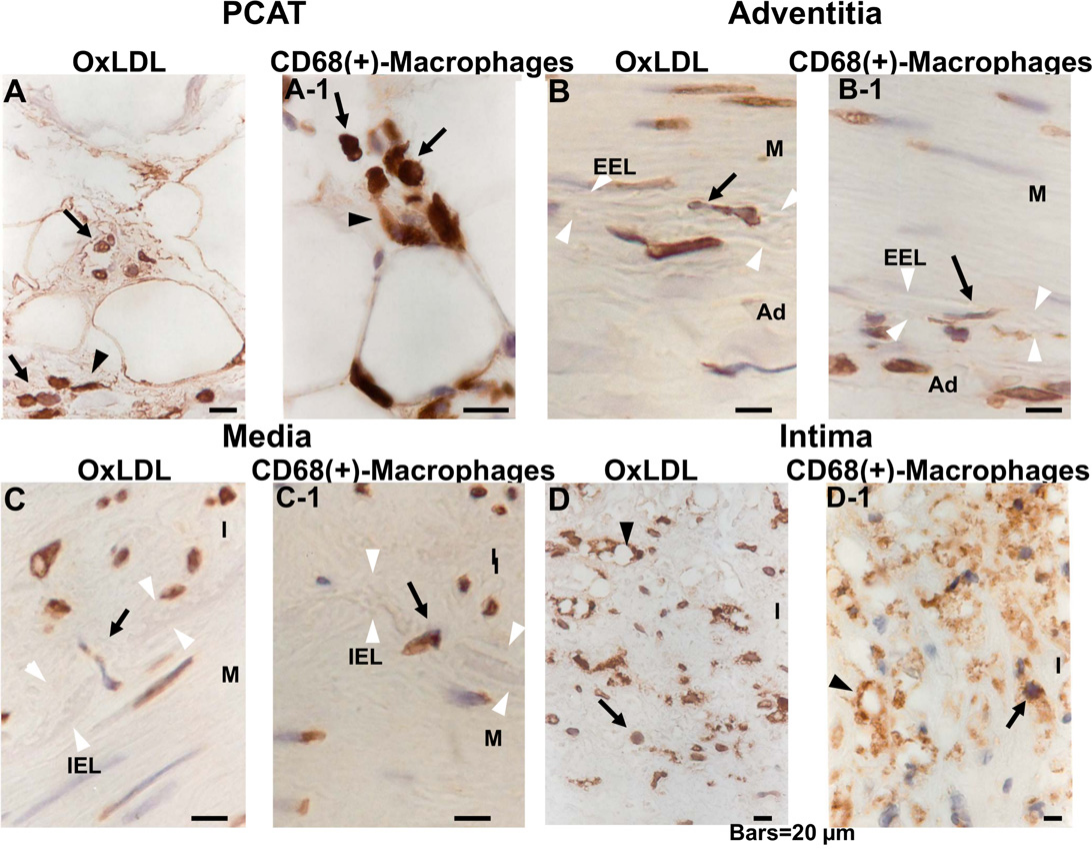
Comparison of dotted oxLDL deposits and CD68 (+)-macrophages. Adjacent sections were stained immunohistochemically for oxLDL or CD68(+)-macrophages for comparison. Both oxLDL deposits and macrophages showed round (arrowhead in A, A-1) or spindle-like configuration (arrows in A, A-1; B, B-1; C, C-1; D, D-1). Dotted deposits of oxLDL and macrophages (arrows in B, B-1, C, C-1) traversing external elastic lamina (EEL; white arrowheads in B, B-1) and internal elastic lamia (IEL; white arrowheads in C, C-1) were demonstrated, strongly suggesting that oxLDL was carried by CD68(+)-macrophages and migrated from adventitia to media, and then to intima. Vacuole-like structures surrounded by oxLDL or CD68 were also observed in intima (arrowheads in D, D-1), suggesting that they are foam cells. Scale9 bars = 20 μm.

Using anti-human oxLDL antibody [17], oxLDL was stained in all PCAT specimens (i.e., epicardial adipose tissue located within 3 mm of an epicardial coronary artery, irrespective of the presence or absence of atherosclerotic plaques, sex and underlying disease at least in the present cases examined “Table 1”. OxLDL was found not only in the cytoplasm but also in the plasma membrane of adipocytes that comprise PCAT “Fig 1A-2 and 1B-2, and Table 2”, indicating a storage function.

**Table 2:** Localization and Deposition Patterns of OxLDL and Their Relation to Plaque Morphology and Vasa Vasorum.

		Normal segments	White plaques	Yellow plaques	
				NC(-)	NC(+)
n = 15	10	25	19		
Incidence					
(a) PCAT		25	19	15	10
(%)		100	100	100	100
					1
(b) Adventitia		24	19	15	14
(%)		96	100	100	100
(c) Media		7*	15	12	6
(%)		28	79	81	60
(d) Intima		8*	16	14†	3
(%)		32	84	93	30

n = number of specimens examined. NC(-): necrotic core absent. NC(+): necrotic core present. PCAT: pericoronary adipose tissue.

* p<0.05 vs PCAT in Normal segments.

† p< 0.05 vs Normal segments and Yellow plaques with NC(+).

### 2. Deposition Patterns of OxLDL in Coronary Intima

OxLDL was deposited in the coronary intima in either a dotted “Fig 1A-1” or diffuse “Fig 1B-1” pattern. In the adventitia and media oxLDL deposited in dotted pattern only, but in either dotted or diffuse, or both patterns in the intima.

### 3. Relationships between Deposition of OxLDL and Plaque Morphology

The percentage (%) incidence of oxLDL in PCAT and adventitia was > 90 irrespective of the presence or absence of coronary atherosclerotic plaques and their morphology “Table 2”. The % incidence of oxLDL in intima was low in normal segments, increased in white (early stage) and yellow plaques without necrotic core (NC; mature stage) and significantly decreased in yellow plaques with NC (end-stage of maturation) that were classified by conventional angioscopy and histology “Table 2”.

### 4. Distribution of Dotted Deposits of OxLDL and CD68(+)-Macrophages

On magnification, dotted deposits were revealed to be mononuclear cells containing oxLDL, in either a round or spindle-like configuration in PCAT, adventitia and intima. Because this configuration resembled that of macrophages, the localization of dotted oxLDL deposits and macrophages (i.e., CD68(+)-macrophages), was compared in adjacent two sections and it coincided almost completely “Fig 2”. OxLDL deposits in a spindle-like or round configuration and also macrophages in similar configurations were observed in the connective tissues (narrow space between the adipocytes) of the PCAT(arrows or arrowheads in Fig 2A and 2A-1), adventitia “Fig 2B and 2B-1”, media “Fig 2C and 2C-1” and intima “arrows in Fig 2D and 2D-1”. In addition, those with the spindle-like configuration were observed traversing the external elastic lamina, which is the border between the adventitia and media “arrows in Fig 2B and 2B-1” and internal elastic lamia, which is the border between the media and intima “arrows in Fig 2C and 2C-1”. Vacuole-like structures circumscribed by oxLDL or CD68 deposits were also observed in the intima, suggesting foam cells, which are well known to be formed from macrophages “arrowheads in Fig 2D and 2D-1” [9].

### 5. OxLDL-containing Macrophages Outside the Vasa Vasorum

**Fig 3:**
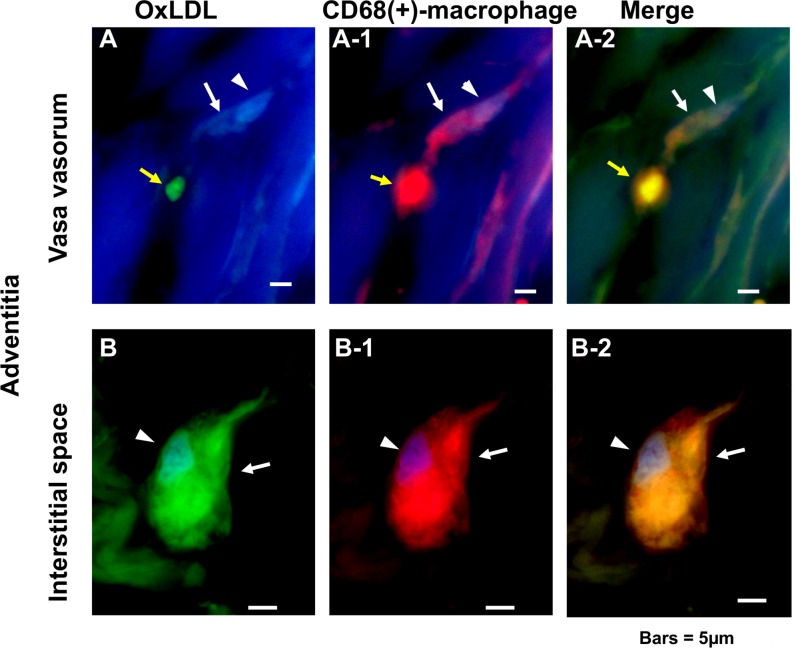
Double immunohistochemical staining of oxLDL and CD68(+)-macrophages in the adventitial vasa vasorum and interstitial space. OxLDL (labeled with FITC) was not contained in CD68(+)-macrophages (labeled with Alexa 555; arrows in A–A-2) inside of adventitial vasa vasorum whereas it was contained in those outside the vasa vasorum (arrows inn B–B-2). Because the macrophage coexisted with a red blood cell (yellow arrows in A–A-2), which is known to exhibit green fluorescence with FITC and red with Alexa 555, its localization inside of the vasa vasorum was confirmed. Arrowheads: nucleus. Scale bars = 5 μm.

**Fig 4:**
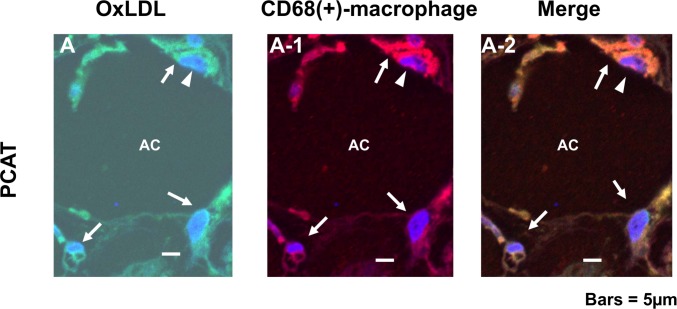
OxLDL-containing CD68(+)-macrophages in pericoronary adipose tissue (PCAT). In PCAT, CD68(+)-macrophages existed in the interstitial space between adipocytes and contained oxLDL (arrows in A—A-2). Arrowheads: nucleus. AC: adipocyte. Scale bars = 5 μm.

Using double immunohostochemical staining of oxLDL and CD68, macrophages within adventitial vasa vasorum were shown not to contain oxLDL “arrows in Fig 3A–3A-2” but those outside these vessels did “arrows in Fig 3B–3B-2”. The macrophages in a spindle-like configuration within the interstitial space of PCAT also contained oxLDL “arrows in Fig 4A–4A-2”.

### 6. Transfer of OxLDL by CD68(+)-Macrophages

**Fig 5:**
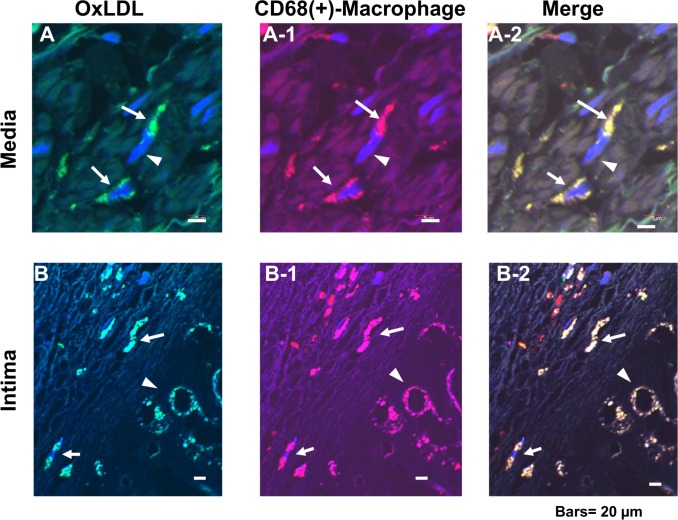
Double immunohistochemical staining of oxLDL and CD68(+)-macrophages in the media and intima. OxLDL-containing macrophages were observed in the media (arrows in A—A-2; arrowheads nuclei) and intima (arrows in B—B-2). Vacuole-like structures contained both oxLDL and CD68 (arrowheads in B—B-2), indicating that these structures are foam cells. Scale bars = 20 μm.

OxLDL containing CD68(+)-macrophages in a spindle-like configuration were also observed in the media “arrows in Fig 5A–5A-2” and intima “arrows in Fig 5B–5B-2”. In addition, vacuole-like structures in the intima were shown to be foam cells because they were positive to oxLDL and CD68 staining “arrowheads in Fig 5B–5B-2”.

### 7. Density of OxLDL-containing Macrophages

There was a good correlation between the PCAT and the intima for the density of oxLDL-containing macrophages, showing Y(density in intima) = 0.82X(density in PCAT) +3.52 (correlation coefficient 0.794, p<0.0001), indicating a close relationship between the oxLDL-containing macrophages in PCAT and those in the intima.

The result of double staining of CD68 and CD31 suggested that CD68(+)-macrophages conveyed oxLDL through the interstitial space of the PCAT, adventitia and media into the intima.

**Fig 6:**
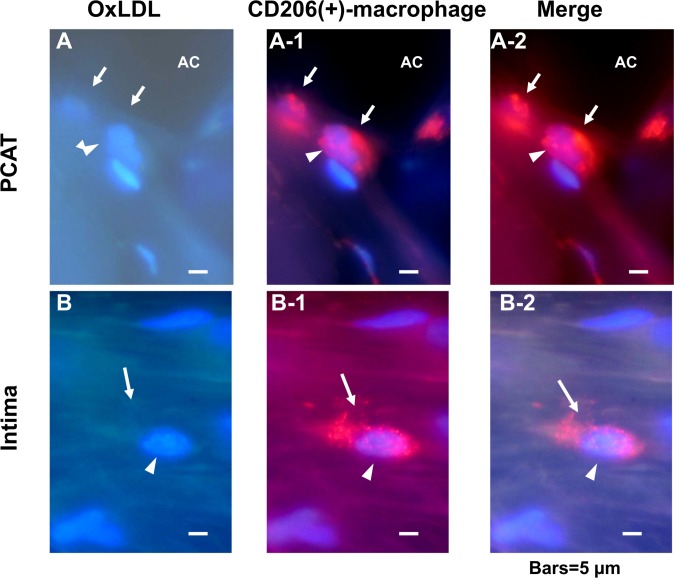
Double immunohistochemical staining of oxLDL and CD206(+)-macrophages in the pericoronary adipose tissue (PCAT) and intima. In the PCAT and intima, oxLDL was not stained (arrows in A, B) but CD206 was stained (arrows in A -1, A-2, B-1, B-2), indicating that CD206(+)-macrophages do not contain oxLDL. Scale bars = 5 μm. AC: adipocyte, Arrowheads: nucleus. Scale bars = 5μm.

In 15 specimens in which dotted oxLDL was found in the intima, oxLDL and CD206, a marker of M2-macrophages, were double stained. CD206(+)-macrophages that contain oxLDL were not found in the PCAT “Fig 6A–6A-2” and intima “Fig 6B–6B-2”.

### 8. Co-localization of Diffuse OxLDL and Intimal Vasa Vasorum

**Fig 7:**
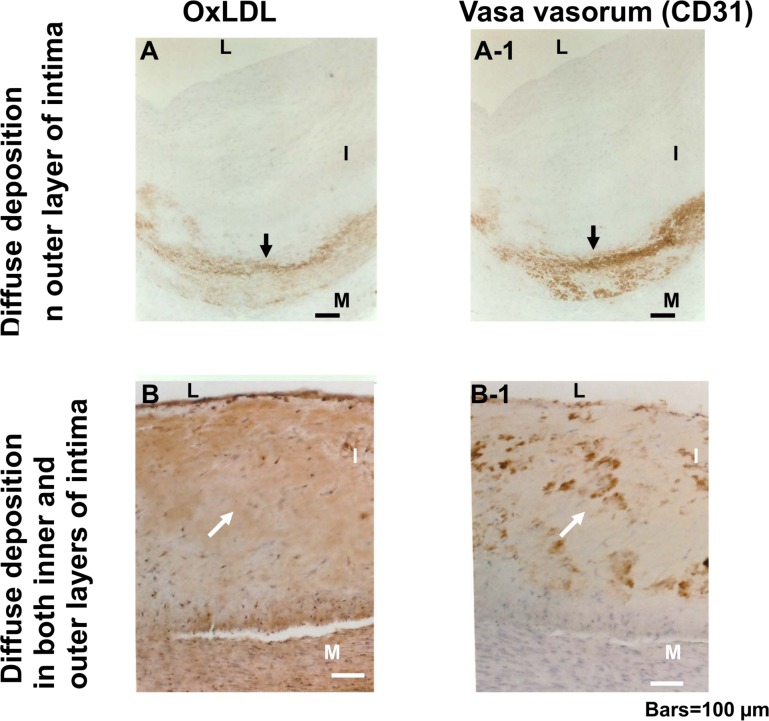
Localization of diffusely deposited oxLDL and vasa vasorum in intima. Diffuse oxLDL deposits (medial side; arrow in A) co-localized with vasa vasorm in the outer half (arrow in A-1), or oxLDL (arrow in B) and vasa vasorum (arrow in B-1) in both inner (luminal side) and outer halves of the intima. Scale bars = 100 μm. L: lumen. I: intima. M: media.

Localization of oxLDL and intimal vasa vasorum (stained by anti-CD31 antibody) was compared in adjacent sections. OxLDL showed a diffuse pattern when intimal vasa vasorum existed “Fig 7 and Table 3”, whereas that in the dotted pattern did not in the majority of preparations “Table 3”.

**Table 3:** Relationship Between Deposition Patterns of Oxidized Low-density Lipoprotein (OxLDL) in the Intima and the Presence or Absence of Intimal Vasa Vasorum.

	Vasa vasorum present	Vasa vasorum absent
Deposition patterns		
of oxLDL		
(a) Dotted deposits	5	17
(b) Diffuse deposits	16*	1

* p< 0.05 vs vasa vasorum absent.

Diffuse oxLDL deposits were localized in outer half (medial side) or in both inner (luminal side) and outer halves of the intima in the majority of preparations. Localization of oxLDL in the intima alone was rare “Table 4”.

**Table 4:** Relationship in Localization Between Diffuse Oxidized low-density Lipoprotein (OxLDL) Deposits and Intimal Vasa Vasorum.

	Localization ofvasa vasorum in intima		
	Outer half	Inner half	Both halves
Localization of			
oxLDL			
(a) Outer half	6	0	2
(b) Inner half	0	2	0
(c) Both halves	1	0	8

## Discussion

Epicardial adipose tissue is mainly composed of adipocytes and an increased volume was recently shown to contribute to the development of atherosclerosis [24]. Perivascular adipose tissue, such as PCAT also belongs, produces large numbers of metabolically active substances with both endocrine and paracrine actions that induce or inhibit atherosclerosis [12]. To date, however, it was not known that PCAT stores and supplies oxLDL, an important pro-atherogenic molecule, to the adjacent coronary arterial intima, which is the site of atherosclerosis as for the perivascular adipose tissue around other vessels.

In the present study, oxLDL deposition in PCAT was observed in all cases, irrespective of the presence or absence of atherosclerotic plaques in the adjacent coronary artery segment or ischemic heart disease as the underlying disease. This finding indicates that oxLDL deposition in PCAT had no relation to the underlying diseases in the cases examined in the present study, indicating the PCAT as a storage of oxLDL, at least in Japanese adults. However it remains to be elucidated whether oxLDL is synthesized in PCAT or supplied from outside, via the adventitial vasa vasorum or penetrating microvessels that directly connect the myocardium with the PCAT.

We observed that macrophages inside the adventitial vasa vasorum did not contain oxLDL, but those outside these vessels contained it in dotted deposition pattern, indicating that oxLDL was not conveyed by macrophages through the vasa vasorum. OxLDL-containing macrophages were observed not only in adventitia, media and intima but also in the PCAT, and those traversing the border of PCAT and adventitia, external elastic lamina (the border of adventitia and media), and internal elastic lamina (the border of media and intima), were also frequently observed. These findings strongly suggested that the macrophages moved from the systemic circulation to adventitial vasa vasorum or through penetrating vessels directly connecting the myocardium and the PCAT, migrated outside these vessels and obtained oxLDL from the PCAT, and conveyed it to the intima through the interstitial space, not through the vasa vasorum, and participated in the development of atherosclerosis.

In the present study, the density of oxLDL-containing macrophages, in the intima positively correlated with that in the PCAT. There are two possibilities for this correlation i.e., oxLDL-containing macrophages in the systemic circulation migrated into both PCAT and intima, or oxLDL-containing macrophages in the PCAT migrated into intima. OxLDL is circulating in the systemic circulation but the macrophages that contain oxLDL in the systemic circulation was not proven [10]. Adventitial vasa vasorum, which connects the systemic circulation via coronary artery, did not contain oxLDL but the macrophages outside the vasa vasorum did. These evidences support the latter mechanism.

The oxLDL also frequently deposited diffusely (not in the dotted pattern) in the outer half, namely the medial side, of intima and co-localized with the vasa vasorum (probably newly formed), indicating that oxLDL alone was conveyed by the vasa vasorum from adventitial side to this portion, but not from the lumen.

The percentage (%) incidence of oxLDL was low in normal coronary segments, increased in white plaque and yellow plaques without NC and decreased in yellow plaques with NC, indicating that oxLDL begins to deposit before plaque formation, increasingly deposits with plaque growth but decreases in the end stage of plaque maturation.

HDL reduces atherosclerosis by facilitating cholesterol uptake from cholesterol-loaded macrophage-foam cells in plaques for transport back to the liver[25, 26] and by decreasing oxLDL [27, 28]. Although definite evidences are lacking, such mechanisms might have participated for the decrease of oxLDL in yellow plaques with NC but not in PCAT, adventitia and media. It seems that there are regional differences in the anti-atherogenic mechanisms. This point remains be elucidated in patients in vivo.

Thus, we obtained the evidences which support that oxLDL is stored in PCAT and conveyed by either CD68(+)-macrophages or neovascularized vasa vasorum into the coronary intima and cause atherosclerosis; i.e., the oxLDL is the key factor that induces the PCAT to act as a risk factor for coronary events in man.

In the present study, CD206(+)-macrophages did not contain oxLDL. Because there are many phenotypes of M2-macrophages (M2a, M2b, M2c, M2d, etc) [18], it remains to be examined whether or not these phenotypes convey oxLDL.

There are a number of macrophage phenotypes. Although it is necessary to confirm which phenotype conveys oxLDL, in clinical situations, however, it is more important to establish a technique to destroy or inhibit the action of the oxLDL-conveying macrophages.

**Fig 8:**
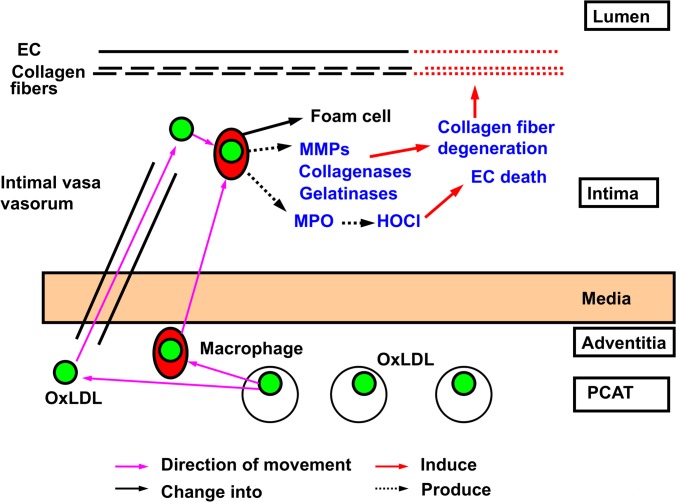
Possible mechanisms for oxLDL supply from pericoronary adipose tissue (PCAT) to coronary intima. Schematic representation of a storage and supply route of oxLDL to the coronary intima. OxLDL is stored in PCAT. The former is conveyed by either macrophages or neovascularized vasa vasorum to the intima, which is the site of atherosclerosis. EC: endothelial cells. MMPs: matrix metalloproteases. MPO: myeloperoxidase. HOCl: hypochlorous acid.

Fig 8 shows our proposal of a PCAT-related mechanism of coronary atherosclerosis based on the findings in the present study. Namely, oxLDL is stored in the PCAT and is supplied to the coronary intima either by CD68(+)-macrophages or vasa vasorum, and participate in atherosclerosis.

## Study Limitations

1. In the present ex vivo immunohistochemical study of oxLDL in human PCAT and adjacent coronary arterial wall, the number of patients belonging to each disease group was very small, and therefore it is not conclusive whether oxLDL deposition in PCAT is related to the underlying disease.
2. The real-time visualization of movement of oxLDL by macrophages or the vasa vasorum was beyond the scope of current immunohistochemical techniques. Therefore, the results from the present study only suggest the supply of oxLDL from the PCAT to the adjacent coronary intima.

## Future Perspectives

Using high-resolution intravascular imaging techniques, such as the intravascular microscope that was devised by us and which can discriminate individual cells down to 10 μm in diameter [29], and biomarkers of oxLDL [30] or by extracorporeal imaging techniques and labeling native oxLDL with isotopes, real-time movement of oxLDL in the vascular wall could be visualized in vivo. Also, movement of oxLDL in PCAT could be visualized in vivo by percutaneous pericardioscopy using a high-resolution endoscope that we also devised [31].

## Conclusions

OxLDL deposition in the PCAT and adjacent coronary arterial wall was investigated ex vivo by immunohistochemical techniques in autopsied adult cases. PCAT stored oxLDL in all study cases, irrespective of the presence or absence of atherosclerosis in the adjacent coronary artery or of the underlying disease.

OxLDL deposited in the coronary intima in a dotted or diffuse pattern. The former represented oxLDL-containing macrophages outside the vasa vasorum and had no relation to the presence or absence of vasa vasorum, its density had a close relation to that in the PCAT. The macrophages traversing the external and internal elastic lamina were frequently observed, strongly suggesting that oxLDL was conveyed by the macrophages from the PCAT to the intima. The oxLDL in the latter pattern colocalized with the vasa vasorum, suggesting that it was conveyed to the intima through neovascularized vasa vasorum.

## Abbreviations

PCAT: pericoronary adipose tissue
NC: necrotic core
oxLDL: oxidized low-density lipoprotein
